# Advancing Quantification of Water-Extractable Arabinoxylan in Beer: A High-Throughput Approach

**DOI:** 10.3390/polym15193959

**Published:** 2023-09-30

**Authors:** Julia Steiner, Michael Kupetz, Thomas Becker

**Affiliations:** Research Group Beverage and Cereal Biotechnology, Institute of Brewing and Beverage Technology, Technical University of Munich, 85354 Freising, Germany

**Keywords:** arabinoxylan, WEAX, quantification, high throughput, acid hydrolysis, xylose, phloroglucinol, recovery rate

## Abstract

Water-extractable arabinoxylan (WEAX) may cause major problems during clarification processes in a brewery owing to its ability to form gel networks. However, high WEAX contents can also enhance the nutritional quality of the final product as they play an important role in the human diet. Therefore, precise quantification of WEAX is required. Current methods are very time- and resource-consuming as well as limited in the number of samples and in some cases provide low accuracy. Thus, a reproducible high-throughput method for the quantification of WEAX optimized for beer was developed, reaching recovery rates (RRs) of almost 100%. The assay is based on Douglas’s colorimetric method. Hydrolysis was conducted using glacial acetic acid to induce the formation of red color complexes resulting from the interaction between pentose degradation products and phloroglucinol. The method was successfully transferred to a multi-mode microplate reader to minimize the loss of color intensity over time and to obtain a high throughput. By using 96-well plates, up to 40% of the previous analysis time could be saved, and a larger number of samples could be analyzed in one batch. The collected data determined xylose as an optimal calibration standard due to high accuracy and reproducibility. The respective AX control standards showed RR within the range of 95–105% without exception. To validate and show the ruggedness of the modified method, WEAX concentration in seven commercial German beers (e.g., lager, pilsner, wheat beer, non-alcoholic beer) was quantified. Interfering hexose sugars that lead to measurement errors when analyzing samples with high amounts of fermentable sugars (e.g., non-alcoholic beer produced by limited fermentation) were eliminated by *Saccharomyces diastaticus* fermentation. Further investigations were carried out by means of LC-MS in order to obtain additional information about the reddish product in the hydrolyzed samples. In this context, C_16_H_12_O_6_ could be identified as one of numerous condensation products, contributing to the coloring. The collected data showed the impact of diverse factors on the measured AX concentration and helped optimize the experimental procedure for a high sample throughput with precise and highly reproducible results. The proposed quantification method should be primarily used in completely fermented finished beer to emphasize the time aspect. Wort samples and non-alcoholic beer produced by limited fermentation can be also analyzed, but only after fermentation with *S. diastaticus*.

## 1. Introduction

As one of the major non-starch polysaccharides in various types of grains (e.g., barley), arabinoxylan (AX) is of particular interest for the food and beverage industry. These polymers may adversely affect product processing and the final product quality [1,2,3]. However, they play an important role in human health and nutrition. Apart from structure and composition, the dietary fiber content is another determining factor for the nutritional, health and taste value of foods [4,5]. Pentose sugars with (1–4)-linked β-D-xylopyranosyl residues form the backbone of AX. Some β-D-xylopyranosyl residues can be substituted with a varying amount of α-L-arabinofuranosyl residues [6,7,8]. The ratio of arabinose to xylose as well as the pattern and degree of substitution influence the cross-linking ability; these are important solubility characteristics of AX that ultimately affect the viscosity of grain or malt extracts [9,10,11]. So far, AX determination has received little attention in the field of beverage analysis and in the interpretation of technological impact in the brewing process. However, due to its proven viscosity-increasing effects in wort and beer, AX is considered as another key component besides β-glucan that has a negative impact on filterability [12]. Measuring only the viscosity often provides insufficient information about the nature and composition of polysaccharides, which means that a specific method for AX quantification is required. The concentration and structural heterogeneity of AX in commercial beers vary considerably and might be explained by the ingredient selection and the respective beer production methods used [13]. According to the literature, AX levels range between 0.5 and 1.9 g L^−1^ in commercial beer samples, and molecular weights between 150 and 10^4^ Da are reported [14]. Scientific publications prove that not only the overall polysaccharide concentration but also the proportion of certain molecular weights is responsible for process engineering problems within the brewery [15,16]. In addition, both aspects have an impact on sensory characteristics in the final product. Palate fullness, for example, is influenced by the range of molecular weight distribution of specific beer components [14,17]. Determining AX within various molecular weight ranges will result in a sharp rise in the number of samples. Consequently, the quantification of water-extractable arabinoxylan (WEAX) in beer requires the development and validation of a simple high-throughput method accompanied by a high measurement accuracy and reproducibility.

There are several techniques for quantifying AX, of which colorimetric assays and chromatographic methods are the most commonly used. While Englyst and Cummings [18] as well as Cleemput et al. [19] used gas chromatography, Houben et al. [20] investigated their samples using liquid chromatography. After hydrolysis of AX with hydrochloric acid, xylose and arabinose were quantified by high-performance anion-exchange chromatography (HPAEC). The structural properties of AX are mainly determined by enzyme mapping [21], molecular weight distribution [22], Fourier transform infrared spectroscopy, Raman spectroscopy, fluorescence microscopy, carbohydrate-binding molecules [23] and gas chromatography (GC-FID) [24]. The colorimetric determination of AX concentration according to Douglas [25] using phloroglucinol traces back to the first description by Wheeler and Tollens [26]. In their studies, they concentrated on the investigation of xylose obtained from wood by acid hydrolysis. When xylose is exposed to phloroglucinol under certain conditions, the color of the sample turns red because of a condensation reaction between the dye and sugar degradation products [26]. This knowledge was later used for the quantification of pentosan by means of acid hydrolysis [27] and formed the basis for the further development of the Douglas method. In 1925, Chase [28] studied the condensation reaction between phloroglucinol and furfural, the main heat-induced degradation product resulting from AX breakdown. According to the literature, different pathways are possible concerning the reaction between phloroglucinol and furfural [28]. However, it has not yet been possible to find a key reaction that is responsible for the formation of the red complex. Until 1957, furfural was distilled at a controlled rate and determined either calorimetrically with orcinol/iron reagent or by gas–liquid chromatography [29]. For reasons of time saving and needing specific equipment, the intermediate distillation step for the enrichment of furfural was eliminated, and the focus was set on the color reaction [30]. The phloroglucinol method applied by Tollens [31] in its original form did not appear expedient for quantitative AX determination, due to strong interference by other sugars and the instability of the color complex. Finally, modifications by Dische [30] and Cracknell [32] generated an amended method that is pentosan-specific in spite of the presence of larger amounts of hexoses [25,33]. The interfering effects were eliminated by reading absorbance at two different wavelengths. The red/pink color complex derived from arabinose and xylose achieved the maximum absorbance at 552 nm but absorbed poorly at 510 nm, whereas the red/orange color complex derived from glucose had the same absorbance at 552 nm and 510 nm. Therefore, the difference in absorbance between 552 and 510 nm led to precise results [34]. Alternatively, Hashimoto [35] and later on Delcour [36] limited the influence of interfering hexoses in hydrolyzed wheat flour samples by yeast fermentation. Even today, several methods are used concurrently for the determination of AX in cereal flours. However, numerous researchers have adopted Douglas’s phloroglucinol method [25] for application to a wide range of cereal-based products [1,2,37,38,39,40] as it has proven to be among the most efficient for AX quantification and is additionally quick and easy to perform. Thus, it was chosen as the basic method for the high-throughput approach.

As the change in color over time and the corresponding loss of absorbance represent one of the major disadvantages, a closer look was taken at the reddish hydrolysate. For a better understanding of the reaction that occurs when heating AX in the presence of phloroglucinol in strongly acidic solutions, the resulting condensation products were analyzed in more detail. The investigation of single substances contributing to the coloring using liquid chromatography (LC) in combination with mass spectrometric detection (MS) was carried out for the first time. Promising compounds that show an absorbance at 550 nm and are thus worth considering for the coloring of the hydrolysates should be identified and further investigated to learn more about the change in color and intensity over time.

The novelty of the methodological approach was the transfer of the Douglas method from a reference-beam photometer to a multi-mode microplate reader to counteract the instability of the colored reaction product and to obtain the necessary high throughput of samples to save time and costs. In order to evaluate the advanced application, several parameters were modified and validated by means of recovery rate and repeatability. Targeted changing of single key factors of the method should eliminate sources of error and optimize the experimental procedure. Due to a strong discrepancy when determining AX content in wort and beer, the aim of the present research was to develop a fast and simple but precise high-throughput approach with a high reproducibility and recovery rates at a constantly high level.

## 2. Materials and Methods

### 2.1. Sample Materials

#### 2.1.1. Calibration and Control Standards

D-(+)-xylose (X1500, purity: ≥99% (dry weight basis)) and L-(+)-arabinose (A3256, purity: ≥99% (dry weight basis)) were purchased from Sigma-Aldrich (Darmstadt, Germany). Wheat AX (P-WAXYM, medium viscosity, *M_w_*: 323 kDa, purity: ~95% (dry weight basis), arabinose: 38%, xylose: 62%) was obtained from Megazyme (Bray, Ireland). All three substances were evaluated as calibration standards. AX was additionally used as a control standard to determine the recovery rate (RR).

To prepare the stock solution for the dilution series of the calibration standards, 100 mg of powdered xylose, arabinose or AX was weighed and diluted in 100 mL of distilled water. Only the AX suspension was heated in a water bath up to 80 °C for 20 min. To ensure thorough mixing, it was homogenized for 1 min. Subsequently, the solution was cooled to room temperature. Dilution series of all calibration standards were produced (0, 50, 100, 150, 200, 250, 300 mg L^−1^). Additionally, two AX control standards (250 and 500 mg L^−1^) were used for each series of measurements to determine RR.

#### 2.1.2. Reaction Reagent Preparation

One reaction reagent according to Douglas [25] and Kiszonas [41] was prepared for sample hydrolysis: 110 mL of glacial acetic acid (100%) was mixed with 2.0 mL of hydrochloric acid (37%); next, 2.0 g phloroglucinol was dissolved in 10.0 mL of absolute ethanol and then added to the acidic solution; mixing ratio 1:5 (sample/reagent). All chemicals were obtained from Merck (Darmstadt, Germany).

#### 2.1.3. Commercial Beer Samples

To validate the transfer to the multi-mode microplate reader for a high sample throughput and high RR, the WEAX concentration in different beer styles (e.g., lager, pilsner, wheat beer) produced in Germany according to the purity law was analyzed. Seven commercial beers with different chemical compositions were chosen: bottom-fermented, barley-malt-based beer: lager (German-style Helles) *n* = 2, pilsner (German-style Pilsner) *n* = 3; top-fermented, barley/wheat-malt-based beer: wheat beer (German-style Hefeweizen) *n* = 1, non-alcoholic wheat beer produced by thermal dealcoholization *n* = 1.

For validation of maximum measurement accuracy and reproducibility of the advanced high-throughput approach to quantify WEAX in beer, a bottom-fermented, barley-malt-based German-style pilsner was chosen.

In the case of high limit dextrin contents, *Saccharomyces diastaticus* (S22, Chair of Brewing and Beverage Technology TUM, Freising, Germany) was added to five different commercial beers that were produced in Germany according to the purity law to reduce potentially interfering hexose sugars. The following beer samples were chosen: bottom-fermented, barley-malt-based beer: lager (German-style Helles) *n* = 1, pilsner (German-style Pilsner) *n* = 1; top-fermented, barley/wheat-malt-based beer: wheat beer (German-style Hefeweizen) *n* = 1, non-alcoholic wheat beer produced by thermal dealcoholization *n* = 1, non-alcoholic wheat beer produced by limited fermentation *n* = 1. Each sample was diluted with distilled water as required for the method of analysis.

### 2.2. Quantification of WEAX by Acid Hydrolysis and Colorimetry

#### 2.2.1. Basic Method According to Douglas and Kiszonas

The colorimetric quantification is based on the phloroglucinol method according to Douglas [25]. Glacial acetic acid is used to break down the AX molecules and further convert the released individual sugar molecules into furan derivatives. These degradation products derived from arabinose and xylose interact with phloroglucinol and result in the formation of red-colored compounds whose absorbance is measured with a spectrophotometer. In consideration of the adjustments of Kiszonas [41], the Douglas assay was modified and conducted using the following procedure: The dilution series of the calibration standard and the AX control standards (AX250 and AX500) were prepared according to Section 2.1.1. For this method of analysis, each beer sample and the AX control standards were diluted 1:8 with distilled water resulting from the assumed WEAX content. An aliquot of 1.0 mL each was transferred to brown glass tubes. Then, 5.0 mL of the freshly prepared reaction reagent was added and the tubes were heated in a water bath (100 °C) for 25 min. Subsequently, all tubes were moved into an ice bath for faster cooling to room temperature. The absorbance of each sample was measured at 550 and 505 nm. To eliminate interference by glucose, the difference in absorbance was calculated. In accordance with the findings of Kiszonas [41], the original addition of glucose to the reaction reagent has proven to be unnecessary as it had no effect on the absorbance values and was therefore eliminated. For the extrapolation of AX concentrations, a second-order non-linear regression curve was used on the basis of the seven-point calibration of the corresponding standard. The resulting polynomial equation was used to convert the absorbance difference into the particular AX concentration. As this procedure does not consider the molecules of water released during hydrolysis, AX values must finally be multiplied by a correction factor of 0.88 according to Hashimoto, Shogren and Pomeranz [35]. All analyses were performed at least in triplicate and in batch mode.

#### 2.2.2. Measurement of Absorbance Using a Spectrophotometer

Samples were prepared and hydrolyzed using the basic method described in Section 2.2.1. Following acid hydrolysis, the samples were analyzed using a conventional reference-beam spectrophotometer (DR 5000 UV-VIS, Hach Lange GmbH, Berlin, Germany). Each sample (including the dilution series of the calibration standard and the AX control standards) was filled into a 10 mm clear cuvette (Merck, Darmstadt, Germany). Since the absorbance had to be read at 550 and 505 nm, the measurement was divided into two parts to avoid changing the wavelength at the spectrophotometer after each sample. During the first round, all samples were measured successively at 550 nm; during the second round, they were measured at 505 nm. In addition, a zero calibration with distilled water in the same cuvette was carried out prior to each single measurement. All samples were assayed in triplicate (*n* = 3 × 2).

#### 2.2.3. Measurement of Absorbance Using a Multi-Mode Microplate Reader

Samples were prepared and hydrolyzed as described in Section 2.2.1. Subsequently, the hydrolysates were analyzed using a hybrid multi-mode microplate reader that combines two double-grating monochromators with a filter-based detection. To perform the measurement, samples were transferred in quadruplicate to a clear 96-well plate, each well filled with 300 µL. Another 300 µL of distilled water was pipetted into one cavity, serving as a blank sample for zero calibration. The value of the blank sample was subtracted from the value of the other samples to eliminate the self-absorption of the solvent. The absorbance was recorded at 550 and 505 nm within a few seconds using the BioTek Synergy H4 with Hybrid Technology (BioTek Instruments, Inc., Winooski, VT, USA). Owing to the time required for pipetting, the absorbance was measured at the earliest 10 min after removing the tubes from the boiling water bath, and the data were analyzed using BioTek Gen5™ Version 2.0 Data Analysis Software. All samples were assayed in triplicate (*n* = 3 × 2 × 4).

#### 2.2.4. Absorbance Loss over Time of the Colored Reaction Product

Since the instability of the color complex has a negative effect on the accuracy and repeatability of the AX determination, the loss of absorbance over time was examined more closely. Calibration and control standards were prepared, and measurements were taken using the multi-mode microplate reader (Section 2.2.3.) to determine the degree of loss of color and intensity. The absorbance was measured for the first time 10 min after removing the tubes from the boiling water bath. Following this, seven further measurements were taken at an interval of 5 min each to demonstrate the extent of absorbance loss over time of the colored reaction product. To determine the optimal timing in view of the multi-mode microplate reader, the RR of the control standards AX250 and AX500 was calculated for each measurement time point. All trials were performed in triplicate (*n* = 3 × 2 × 4).

#### 2.2.5. Validation of the High-Throughput Approach

Single laboratory validation was performed by measuring the AX concentration of one commercial beer (i.e., pilsner) and the two control standards (AX250 and AX500). Sensitivity, ruggedness, precision, standard preparation and measurement inaccuracy were considered to validate the method for AX quantification in the studied beer samples. To ensure high reproducibility and repeatability, the AX concentrations of the beer sample and the control standards were measured numerous times. Calibration and control standards as well as five replicates of the beer sample from the same batch but different bottles were prepared in separate tubes and hydrolyzed simultaneously (Section 2.2.1). All samples were transferred to the same plate in order to read the absorbance at the same time (*n* = 5 × 2 × 4). Measurements were carried out exactly 10 min after removing the tubes from the boiling water bath using the multi-mode microplate reader according to Section 2.2.3. All trials were performed in triplicate (*n* = 3 × 40). The same procedure was performed in total on five different days, each day with fresh beer samples from a different batch. For statistical analysis, the mean value, variance and standard deviation were calculated. Outliers were checked and removed based on the Grubbs test. To determine the significance of differences between the values, one-way analysis of variance (ANOVA) was used, followed by Fisher’s least significant difference test with a significance level (α) of 0.05.

### 2.3. Elimination of Interfering Sugars with Saccharomyces diastaticus Fermentation

*S. diastaticus* yeast was added to five different commercial beers to metabolize potentially interfering hexoses in the samples with high amounts of limit dextrin and fermentable sugars. To verify the reduction of glucose molecules, 150 mL of a non-alcoholic wheat beer produced by limited fermentation was inoculated with 1.0 mL yeast suspension and fermented in an incubation chamber (37 °C) for several days. To confirm that this yeast strain does not metabolize AX, two wheat and two lager beers with a small amount of limit dextrin and remaining fermentable sugars were treated following the same procedure. Immediately after inoculation, samples were taken periodically at an interval of 24 h. Samples were prepared and hydrolyzed as described in Section 2.2.1, and absorbance was measured using the multi-mode microplate reader according to Section 2.2.3. All trials were performed in triplicate (*n* = 3 × 2 × 4).

### 2.4. Investigation of the Red Color Complex by Liquid Chromatography

For the formation of the pink/red product that results when hydrolyzing pentose sugars in the presence of phloroglucinol, only xylose standard material was used. Samples were analyzed by means of LC-MS before and immediately after acid hydrolysis. Due to color loss over time, samples with a xylose concentration of 2 g L^−1^ were used to create a more intense color for the identification of the chromophoric product. Samples were prepared and hydrolyzed as described in Section 2.2.1. All analyses were performed in triplicate (*n* = 3).

#### 2.4.1. High-Performance Liquid Chromatography (HPLC)

High-performance liquid chromatography was used for peak separation in order to characterize potential components from the elution profile detectable at 550 nm. The system consisted of an UltiMate 3000 Autosampler, an UltiMate 3000 pump module, an UltiMate 3000 column compartment and an UltiMate 3000 Diode Array Detector (Thermo Fisher Scientific Inc. Waltham, MA, USA). As the stationary phase, a Kinetex column (2.6 μm C18, 100 Å, 150 × 2.1 mm Phenomenex, Aschaffenburg, Germany) was used. Solvent A (water + 0.1% (*v*/*v*) formic acid) and Solvent B (acetonitrile + 0.1% (*v*/*v*) formic acid) were mixed in gradient mode (0–0.5 min 80% A + 20% B; 1–10 min linear gradient to 100% B; 10.5–13 min 100% B; 13.5–15 min 80% A + 20% B). The flow rate was 0.5 mL min^−1^, the injection volume was 50 μL and the detection wavelength was 550 nm. Data evaluation was performed using Chromeleon 6.80 Software from Thermo Fisher Scientific Inc.

#### 2.4.2. Liquid Chromatography-Mass Spectrometry (LC-MS)

For the identification of the colored compounds and verification with regard to the literature, liquid chromatography was additionally coupled with mass spectrometry. The HPLC Agilent 1200 series system (Agilent, Waldbronn, Germany) consisted of a HiP-ALS SL autosampler, a 1200 series bin pump module, a 1200 series degasser and an 1100 series column oven coupled with an UltiMate 3000 MWD (Thermo Fisher Scientific Inc.) and a Triple Quad 4500 MS (Sciex, Darmstadt, Germany). The ion spray voltage was set to 5500 V, and full scan mass spectra were measured between mass-to-charge ratio *m*/*z* 50 and 2000 in positive ion mode. The stationary phase was a Kinetex 2.6 μm, 150 × 2.1 mm column that was used with water with 1% (*v*/*v*) formic acid as Solvent A and acetonitrile with 1% (*v*/*v*) formic acid as Solvent B. The flow rate was 1.0 mL min^−1^, and the gradient mode was as follows: 0–0.5 min 80% A + 20% B; 1–15 min linear gradient to 100% B; 16–19 min 100% B; 19.5–25 min 80% A + 20% B. Samples were filtered (0.45 μm); the injection volume was 50 μL, and the detection wavelength was 550 nm. All measurements were performed in triplicate (*n* = 3), and data evaluation was performed using Analyst 1.6.3 Software from Sciex.

### 2.5. Statistical Analysis

The results were statistically evaluated using OriginPro 2015G (OriginLab Cooperation, Northampton, MA, USA). To determine the significance of differences between the values, one-way ANOVA was used, followed by Fisher’s least significant difference test with a significance level (α) of 0.05.

## 3. Results and Discussion

### 3.1. Improvement of the Colorimetric Quantification of WEAX

The development of a high-throughput approach must considerably improve the reproducibility and accuracy of the colorimetric assay and ensure the analysis of a large sample number. So far, AX determination has received too little attention although it is considered as another technological as well as nutritional parameter besides β-glucan. Until now, it has been difficult to assess the role of the AX substance group since no sufficiently rapid and precise standard measurement methodology was available. Breweries should use this application for better predictability and interpretation of corresponding parameters with regard to raw material input, individual processing steps and the finished product.

#### 3.1.1. Spectrophotometer vs. Multi-Mode Microplate Reader

Calibration and control standards as well as commercial beer samples were prepared and hydrolyzed using the basic method described in Section 2.2.1. To minimize the absorbance loss when using spectrophotometry and to ensure a high sample throughput, the intention was to transfer the measuring procedure to the multi-mode microplate reader to determine the absorbance of all standards and samples at the same time. Initially, measurements were taken using the reference-beam photometer at two different wavelengths. With an additional zero adjustment before each sample, the overall measuring procedure was highly time-consuming. Furthermore, the pink/red reaction product is extremely sensitive to light, leading to a color and intensity change over time. Particularly, the loss of intensity that results in an absorbance decrease may negatively affect the accuracy and reproducibility of AX quantification. Regarding the multi-mode microplate reader, the time factor during the measurement itself can be neglected as all samples are read simultaneously. In this case, the overall reading duration including the change in wavelength takes less than 30 s. However, the measurement time point (t_m_) is shifted backward owing to the time interval (Δt) required for the transfer of the samples to the plate.

**Figure 1:**
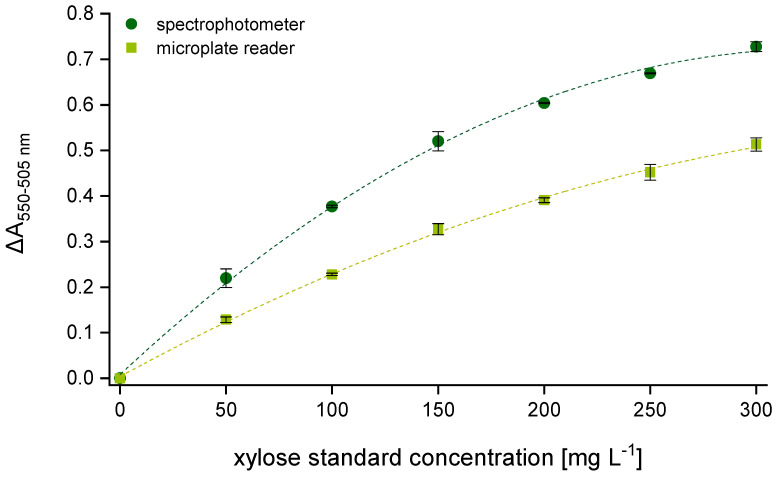
Difference in absorbance (ΔA_550–505 nm_) as a function of the weighed xylose concentration (0, 50, 100, 150, 200, 250, 300 mg L^−1^). Comparison of two calibration curves obtained using two different photometric systems (*n* = 3). Legend: 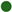 spectrophotometer, 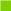 multi-mode microplate reader.

Figure 1 shows two calibration curves (seven-point calibration) as they are used for AX quantification according to Douglas [25]. The absorbance values that are needed to obtain the corresponding calibration curve result from reading the hydrolyzed samples by means of the two mentioned spectral measuring principles. Both graphs were generated from a second-order polynomial fit by associating the absorbance difference between 550 and 505 nm (ΔA_550–505 nm_ = E_550_ − E_505_) with the respective concentration of the xylose dilution series. Although the same samples were measured, the two calibration curves have considerably different shapes described by the following equations: y = −6.598 × 10^−6^x^2^ + 0.004x + 0.008 (R^2^ = 0.998) for the spectrophotometer and y = −2.858 × 10^−6^x^2^ + 0.002x + 0.004 (R^2^ = 0.998) for the multi-mode microplate reader.

The ΔA_550–505_ values that result from the analysis via spectrophotometer are significantly higher compared to those measured by means of the multi-mode microplate reader. Due to lower absorbance values at 505 nm, the difference in absorbance between 550 and 505 nm becomes substantially larger. During the time (Δt_550_) elapsing while reading all samples at 550 nm one after another, the color intensity has already decreased, which leads to a clear absorbance loss when measuring the first sample at 505 nm. The interval can vary depending on the number of samples provided. When analyzing only one beer sample, seven calibration standards and two AX control standards, Δt_550_ amounts to approximately 10 min. With each additional sample, the interval increases by one minute, which might have a distorting effect on the resulting AX values.

**Figure 2:**
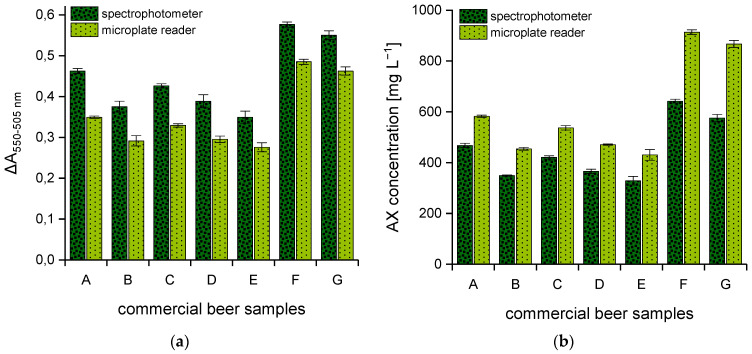
Difference in absorbance (ΔA_550–505 nm_) of seven commercial German beer samples from various breweries determined using two different photometric systems (*n* = 3) (**a**). AX concentrations [mg L^−1^] were calculated from the corresponding calibration curve (**b**). Legend: 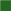 (dark green) spectrophotometer, 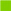 (light green) multi-mode microplate reader. Beer samples: lager (A), lager (B), pilsner (C), pilsner (D), pilsner (E), wheat beer (F), non-alcoholic wheat beer (G).

For further investigations, seven commercial German beers and the two AX control standards were analyzed using both spectral systems. All samples were prepared in duplicate according to Section 2.2.1, and aliquots from each tube were taken for absorbance reading according to Section 2.2.2 and Section 2.2.3. The respective AX content was calculated from the polynomial equation of the corresponding calibration curve. Comparable to the xylose calibration standards (Figure 1), absorbance differences of the beer samples that result from the analysis via spectrophotometer are likewise significantly higher than the values measured by means of the multi-mode microplate reader (see Figure 2a). In contrast, AX concentrations that result from measurements with the spectrophotometer were 20–35% lower with a maximum measuring error of ±20% in comparison to those obtained using the multi-mode microplate reader (see Figure 2b).

Additionally, the AX control standards both reached an RR of 100 ± 2% when the hydrolysates were measured with the multi-mode microplate reader. Using the spectrophotometer, the RR of the two control standards showed noticeable fluctuations up to 25% in both directions and only occasionally reached the acceptable range between 90 and 110%. Since the spectrophotometer showed some disadvantages for the measurement of AX, the multi-mode microplate reader was used for further detailed investigations.

In order to demonstrate the scope of the quantification method, the seven commercial beers were selected from a variety of top- and bottom-fermented samples with different chemical compositions. Since the ingredients in German beer production are strictly regulated and, above all, the use of enzymes is not permitted, the AX content could have greater relevance, especially with regard to the molecular weight distribution. For this reason, beers produced according to the German Purity Law were explicitly selected. AX concentrations ranged between 430.5 and 913.9 mg L^−1^. The fluctuations in AX concentrations (see Figure 2b), especially within one beer style (i.e., samples A and B) are traced back to the raw materials (e.g., malt) but also, of course, to differences in the production process. However, as soon as wheat malt is additionally used, the AX contents increase significantly, since the AX content in unmalted wheat grain already implies significantly higher values.

#### 3.1.2. Determination of the Optimum Measurement Time Point Using Multi-Mode Microplate Reader

**Figure 3:**
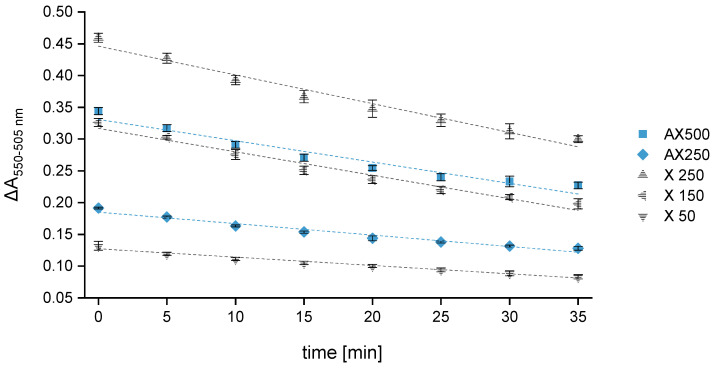
Variation in the difference in absorbance (ΔA_550–505 nm_) over time for three xylose calibration standards (X: 50, 150, 250 mg L^–1^) and two control standards (AX: 250 and 500 mg L^–1^). Absorbance was measured every 5 min over a period of 30 min (*n* = 3). Legend: 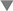 X 50, 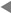 X 150, 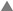 X 250, 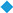 AX250, 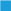 AX500.

A great advantage attributed to the multi-mode microplate reader is that all samples and standards are included in one plate and measured simultaneously. This reduces the recording period at the respective wavelength to only 10 s. Switching from 550 to 505 nm takes less than 5 s. As the overall measurement duration is very short (<30 s), the time factor during the reading process can be excluded as a source of error. Since 96-well plates are used, fourteen samples plus calibration and AX control standards can be measured per experimental series in quadruplicate (*n* = 4). Compared to the reference-beam spectrophotometer, considerably more samples can be analyzed in a significantly shorter time, leading to a significantly higher sample throughput. The main disadvantage of a 96-well plate is the time needed for sample transfer, which takes about 8 to 10 min. Thus, the measurement can be started at the earliest 10 min after removing the samples from the boiling water bath. To obtain results that are as accurate and reproducible as possible, this 10 min time interval was examined more closely. The analyses have shown that the difference in absorbance of the control standards AX250 and AX500 decreases on average by 15% when measuring the same samples immediately (t_m_ = 0 min) and again after 10 min (see Figure 3). When calculating the absolute AX concentrations of the control standards using the calibration curve constructed from the absorbance values measured at t_m_ = 0 min, it could be observed that a time span of 10 min results in even lower AX values (reduction of 18–20%). However, if the corresponding calibration curve constructed from the absorbance values measured at t_m_ = 10 min is used as a calculation basis, the absolute AX concentrations of the control standards only deviate by up to 3%, which is within the range of error tolerance. The absorbance differences of the xylose calibration standards measured at t_m_ = 10 min (see Figure 3) show a decrease of approximately 15 ± 1% compared to the values measured at t_m_ = 0 min. Therefore, the absorbance losses of the AX control standards are evidently compensated and have no significant impact on the final calculated concentrations. However, since all samples are on the same plate, there is no time lag between the measurement of the calibration standards and the control standards.

Two control standards (AX250 and 500) and three calibration standards with different xylose concentrations (50, 150, 250 mg L^−1^) were monitored over a period of 35 min, and the absorbance of each sample was measured every 5 min. A linear regression model relating the absorbance difference (ΔA_550–505_) to the time was used. A_550_ is much higher compared to A_505_ but has a noticeably greater loss rate accordingly leading to a constant decrease in the absorbance difference. Independent of the concentration, all samples showed the same loss rate, entailing a reduction of about 35 ± 3% after 35 min. Consequently, the standard with the highest xylose concentration showed the greatest loss of the absorbance difference over time. Furthermore, it could be observed that the slope of the straight line begins to flatten after half an hour, which may potentially lead to a falsification of the calculated AX values when deferring the measurement time by more than 30 min. Similarly, Kiszonas’s investigations [41] showed a constant decrease in absorbance over time. However, the loss rates were noticeably smaller. Different from our experimental setup, a reference-beam spectrophotometer was used by Kiszonas.

To investigate the influence of the time shift (Δt) on the calculated AX concentration, the RR of the two control standards was determined. Reading the absorbance immediately after the sample transfer to the plate and calculating the AX concentration using the corresponding calibration curve resulted in an RR within the error tolerance level of 95 to 105%. Further measurements after 10, 20 and 30 min delivered an RR still within an acceptable range between 90 and 110%. A measurement delay of more than 30 min occasionally led to an increase in the RR to 115 ± 5%, surpassing the target.

The fact is that the time interval Δt, which includes sample cooling and transfer time, cannot be shortened any further, so the first reading can only be taken at the earliest 10 min after hydrolysis. Regardless, it was found that when reading the absorbance 10 to 12 min after the samples were removed from the water bath, an RR within an absolute range of 95–105% was obtained for more than 90% of all measurements (*n* = 50) of the AX control standards. With a maximum sample transfer time of 10 min, the cooling time was therefore set to 2 min to achieve an appropriate time interval (Δt_max_ = 12 min). Even with a smaller sample number, which shortens the transfer time, the measurement of absorbance should also be started only after 10 min to ensure comparability and reproducibility. Using a correction factor based on the loss rate to compensate the decrease in absorbance as Kiszonas, Courtin and Morris [41] suggest could falsify the calculated results.

#### 3.1.3. Comparison of Standard Material for External Calibration Using Multi-Mode Microplate Reader

For further optimization, arabinose, xylose and AX were examined as possible reference substances for external calibration. Overall, the individual standard materials showed no significant deviation (*p* > 0.05) in the difference in absorbance at any concentration. The respective AX concentrations of the controls showed an RR in a range between 95 and 105% without exception. These findings are in agreement with Kiszonas’s observation [41] that arabinose and xylose produce identical absorbance response curves, so either could be used as a calibration standard. Additionally, we were able to demonstrate that the two monosaccharides could also be replaced by AX. Based on our results, xylose has emerged as an optimal model standard. Not only the high RR and the good reproducibility but also the properties during sample preparation and price were deciding factors.

#### 3.1.4. Reproducibility of the Method Using Multi-Mode Microplate Reader

For validation of the high-throughput method, a multiple determination of one commercial lager beer and the two control standards (AX250 and AX500) was carried out. The determination of the AX concentration was performed in batches, measuring five replicates of the pilsner beer sample together with the xylose calibration standards on a 96-well plate. The same procedure was repeated four times on five different days. A multiple determination on the same plate and the measurement of the same samples on various days showed no significant difference (*p* > 0.05). The lager beer exhibited a concentration of 695.8 mg L^−1^ (SD = 41.4 mg L^−1^; 95% CI [644.5, 747.3 mg L^−1^]). The mean value of AX250 was determined as 238.5 mg L^−1^ (SD = 11.9 mg L^−1^; 95% CI [225.9, 251.1 mg L^−1^]), while in AX500, an average content of 516.8 mg L^−1^ (SD = 32.4 mg L^−1^; 95% CI [482.9, 550.8 mg L^−1^]) was measured. For all samples, the relative standard deviation was less than 5%, which indicates a high reproducibility of the results. In a second step, the lager beer sample was spiked with purified AX in two different concentrations (125 mg L^−1^ and 250 mg L^−1^). After hydrolysis according to Douglas, the total amount of AX was determined using the multi-mode microplate reader. When subtracting the mean value (*n* = 50) of AX determined in the control beer (695.8 mg L^−1^), the AX amount added subsequently to the lager beer showed an RR within the error tolerance level of 95 to 105% without exception (*n* = 3 × 2 × 4).

### 3.2. Influence of S. diastaticus Fermentation on the Quantification of AX in Samples with Higher Amounts of Interfering Sugars

The modified and validated quantification procedure could not be applied for beers with high amounts of limit dextrin and fermentable sugars without a preliminary treatment, due to the reaction of the dye with degraded hexose sugars. For this reason, *S. diastaticus* yeast was used to metabolize fermentable mono-, di- and oligosaccharides and thus eliminate interferences during absorbance reading. Investigations by Douglas [25] and Rouau [34] have already shown that some sugars may interfere with AX quantification as they form reddish-colored products during heat-induced acid hydrolysis in the presence of phloroglucinol. In this case, glucose and fructose building blocks mainly act as interfering substances. Dische and Borenfreund [30] demonstrated that subtracting the absorbance at 505 nm from that at 550 nm is necessary to allow an accurate quantification of AX, especially when there is an excess of interfering sugars.

**Figure 4:**
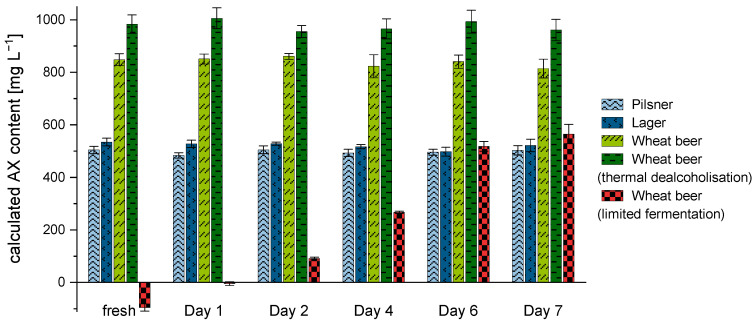
AX concentration [mg L^−1^] (*n* = 3) in different beer types during fermentation with *S. diastaticus* over a period of seven days. Legend: (light blue) pilsner, (dark blue) lager, (light green) wheat beer, (dark green) non-alcoholic wheat beer produced by thermal dealcoholization, (red/black) non-alcoholic wheat beer produced by limited fermentation.

When determining AX concentration in lager beer with a comparable high degree of fermentation, absorbance at 505 nm shows substantially lower values than those at 550 nm. By comparison, the absorbance difference measured in non-alcoholic wheat beer samples (limited fermentation) showed negative values, as the absorbance values at 505 nm were noticeably higher than those at 550 nm. According to the available literature, a negative absorbance response is attributed to an excessively high content of interfering sugars [25,34]. The average sugar content of a non-alcoholic beer (limited fermentation) ranges between 2 and 4 g/100 mL, much higher than that of a conventional lager or wheat beer containing alcohol (sugar content: <0.5 g/100 mL). Since the amount of interfering sugars is too high to be compensated by the difference in absorbance and consequently results in an underestimation of the AX content, these substances have to be eliminated. To investigate the impact of *S. diastaticus* on the reduction of interfering sugars in the corresponding samples, five different commercial beers were inoculated and incubated for several days. Only the non-alcoholic wheat beer sample produced by limited fermentation showed significant differences in AX concentration during the fermentation process (see Figure 4).

In the non-alcoholic wheat beer (limited fermentation), the calculated AX content increased significantly (*p* < 0.05) over time from a negative value up to 565.1 ± 36.6 mg L^−1^. After a period of six days, no further significant increase (*p* > 0.05) of the calculated concentration could be observed in this sample. In comparison, the bottom-fermented beers (i.e., lager and pilsner) did not show any significant difference (*p* > 0.05) in AX concentration over the entire fermentation period. In addition, a thermally dealcoholized wheat beer (<0.5% vol.) and a conventional wheat beer containing 5% vol. alcohol were analyzed. When quantifying AX over seven days of fermentation, observations similar to those for the bottom-fermented beers could be made. The AX values of both wheat beer samples remained constant at 814.6 ± 35.9 mg L^−1^ (conventional wheat beer) and 961.1 ± 40.9 mg L^−1^ (thermally dealcoholized wheat beer) over the fermentation period (*p* > 0.05). These results provide evidence that the use of *S. diastaticus* is able to adjust the underestimation of AX contents by reducing interfering sugars, without affecting the xylose and arabinose response. In the experiments, it could be shown that the fermentation step is absolutely necessary in samples with sugar contents above 3.5 g/100 mL and a total carbohydrate content of 5.0 g/100 mL. Therefore, the use of *S. diastaticus* is recommended for samples that have not undergone fermentation (e.g., wort) or for which the fermentation was incomplete. The measurements (*n* = 3 × 2 × 4) were highly reproducible, with a variation coefficient of <5%.

### 3.3. Investigation of the Red Color Complex by LC-MS

**Figure 5:**
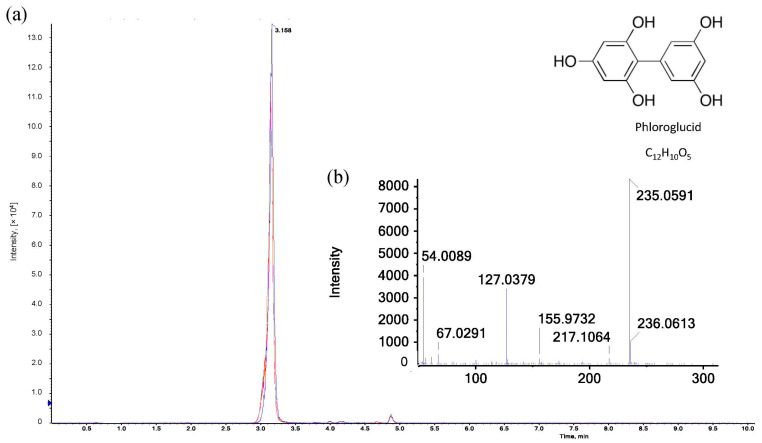
LC-MS analysis of phloroglucide: (**a**) Extracted ion chromatograms (XICs) of three different samples after heat-induced acid hydrolysis with reaction reagent recorded in positive electrospray ionization (ESI) mode (*n* = 3). Legend: 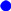 reaction reagent as reference, 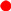 reaction reagent with xylose, 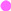 reaction reagent with AX. (**b**) ESI-MS/MS spectrum of [M+H]^+^ ion (*m*/*z* 235.06) of phloroglucide in the hydrolyzed reaction reagent.

In order to obtain more information about the red/pink product in the hydrolyzed samples, further investigations were carried out by means of liquid chromatography. In the first step, the elution profile of ion-exchange chromatography coupled with a diode array detector was examined to identify fractions with compounds active at 550 nm. Absorbance at this wavelength yielded numerous peaks in a coherent area within a retention time window of 5 min. Comparing the elution profiles of three consecutive measurements of the same sample with an interval of 20 min, a significant decrease (*p* < 0.05) in absorbance at 550 nm could be observed, which confirms the findings of the spectrophotometric measurements. Trials were carried out in triplicate (*n* = 3) to ensure high reproducibility of the elution profile with respect to the light-sensitive substances to be investigated. In the next step, the separation module was coupled with a mass spectrometer to identify the compounds that are responsible for the red/pink coloring of the sample. According to Kiszonas [41], the red color is attributed to simple phloroglucide (C_12_H_10_O_5_, M = 235 Da) that results from the heat-induced acid hydrolysis of pentose sugars (see Figure 5). Furfural resulting from hydrolysis donates electrons to two molecules of phloroglucinol, which condense to form phloroglucide [41]. The literature, however, shows that simple phloroglucide (C_12_H_10_O_5_) is already formed without thermal influence only under exposure to concentrated hydrochloric and glacial acetic acid. The strongly acidic solution induces a condensation of two phloroglucinol molecules under the elimination of water. Subsequently, complex phloroglucide derivatives (partially colored) may be formed when diverse chemical compounds are added to simple phloroglucide. These reaction procedures may additionally be favored by high temperatures [42]. Furthermore, (colored) phloroglucide derivatives may also arise from condensation reactions of phloroglucinol with substances that result from the hydrolysis/distillation of samples rich in carbohydrates [43]. Within our investigations, C_12_H_10_O_5_ could already be identified in the freshly prepared reaction reagent that remained colorless even after boiling. The absorbance values at both 550 and 505 nm were equal to zero before and after the pure reaction reagent was boiled. Therefore, simple phloroglucide (C_12_H_10_O_5_) cannot be responsible for the red/pink coloring of the hydrolyzed AX samples, so C_12_H_10_O_5_ is not the chromophoric key product analyzed by the Douglas AX assay.

However, adding xylose or arabinose to the acidic reaction reagent and boiling this mixture for 25 min resulted in red coloration. According to the literature, coloring is caused by complex phloroglucide derivatives detected at a wavelength of 550 nm resulting from the condensation of phloroglucinol with respective furan derivatives [34]. For AX, furfural phloroglucide is the key product investigated in more detail with regard to the red coloring of the distillates. So far, there is no consensus on what the condensation product looks like. Chase [28] outlines earlier investigations and gives an overview of possible reactions between phloroglucinol and furfural assumed by different researchers. Table 1 shows a limited summary of potential condensation products that could be proven relevant and considered to be the most probable. It is probable that more than one substance is responsible for the coloring.

**Figure 6:**
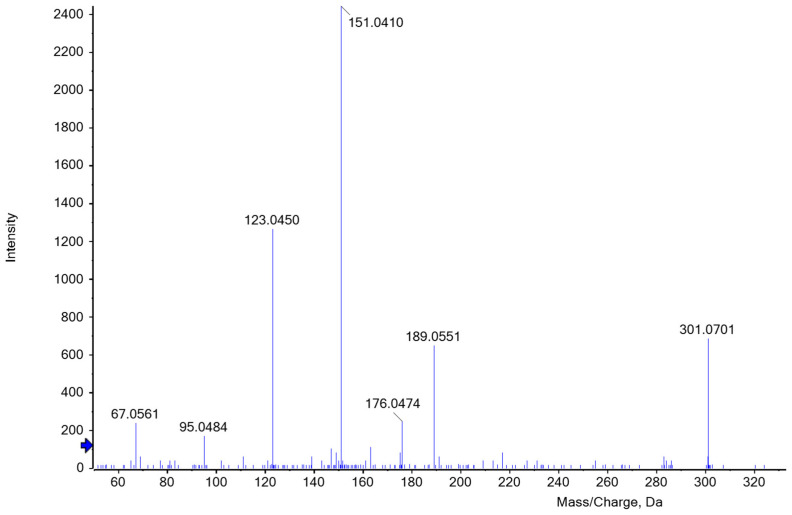
ESI-MS/MS spectrum of [M+H]^+^ ion (*m*/*z* 301.1) of C_16_H_12_O_6_ at a collision energy of 35 eV. C_16_H_12_O_6_ could be one of the most probable furfural phloroglucide derivatives that occur as a red color complex during heat-induced acid hydrolysis of the reaction reagent in the presence of AX.

The identification of the key product was difficult due to interfering substances and the loss of color and intensity. The elution profile of ion-exchange chromatography indicated that several substances were detected at 550 nm. According to the literature, hydroxymethylfurfural derived from hexoses may also turn into a reddish condensation product that has an absorbance maximum at 550 nm [34]. In the distillation of various carbohydrates, Klingstedt [43] observed that furfural and hydroxymethylfurfural are formed with a time shift, which in turn leads to the delayed formation of the corresponding condensation products. Hence, a variation in boiling time may cause a change in the composition of the hydrolysate and its colorimetric properties. To gain an appropriate amount of the chromophoric key product and to ensure high reproducibility when comparing samples, a constant hydrolysis period and the time of measurement are of major importance. Nevertheless, some of the products seem to be extremely unstable and potentially change the structure and properties of the initial molecule within a very short time. Foo and Hemingway [50] suggest that the product C_17_H_14_O_7_ possibly undergoes self-condensation, forming an oligomer (C_28_H_22_O_11_) with the release of phloroglucinol. The potential furfural phloroglucide derivatives listed in Table 1 should be identified based on the calculated *m*/*z* values. According to our mass spectrometry results, one of the substances most likely formed during distillation is C_16_H_12_O_6_, which agrees with Councler [48] and Leach [49]. The proposed structure corresponds to the accurate mass measured for the intact molecule. Figure 6 shows the MS/MS fragmentation of *m*/*z* 301 Da in the positive ion mode after separation by HPLC.

Chase [28] also suggested C_16_H_12_O_6_ and additionally C_22_H_16_O_8_ as the most probable substances resulting from the phloroglucinol furfural reaction. Within his investigations, he observed that the reaction takes place with the enol form of phloroglucinol, significantly reducing options regarding the resulting key products. Moreover, he considered a more complicated reaction of phloroglucinol condensing with itself to form an ether and with the aldehyde group of the furfural resulting as C_17_H_12_O_6_, the acetal of the derivative described by Foo and Hemingway [50]. Several authors even assumed a polycondensation of either linear or cross-linked reaction products. Regarding our mass spectrometric measurements, C_22_H_16_O_8_ and C_17_H_14_O_7_ could be potential products resulting from the condensation reaction between phloroglucinol (P) and furfural (F), the former at a ratio of 2P to 2F and the latter at a ratio of 2P to 1F. When analyzing the elution profile, clear peaks could be observed at *m*/*z* 409 and 331 Da. However, the absolute spectral intensity of the molecular ions is significantly lower than at *m*/*z* 301 although a constant collision energy of 35eV was used. MS/MS fragmentation of the ion at *m*/*z* 401 Da produced a distinctive ion at *m*/*z* 331 Da. In some cases, however, the intensity of the fragment ions was lost in the background noise. Although the colored hydrolysate was fractionated by HPLC, numerous ions are present in the MS spectrum of the red fraction that are not always a result of in-source fragmentation of the possible derivative, but reflect other (interfering) hydrolysis products. Possibly, the ability to screen for suggested compounds present at unknown levels may be hampered by chemical background noise. For this reason, an unambiguous identification of one product was virtually impossible. Even after the completion of our investigations, there are still several substances that should be considered in red coloring. To the best of our knowledge, this is the first report employing high-resolution mass spectrometry for the study of furfural phloroglucide derivatives in samples hydrolyzed in order to determine AX concentration. This work will provide a helpful basis for further study of different phloroglucide derivatives of colorimetric assays to understand color formation in dependence on hydrolysis conditions better.

## 4. Conclusions

Based on the collected data, it can be concluded that the use of a multi-mode microplate reader in combination with the adjustment of selected parameters considerably improved the reproducibility and accuracy of the colorimetric assay, with an RR of nearly 100%. The calculated AX concentrations showed small standard deviations with a maximum variation coefficient of 5%. The difference in absorbance shows a linear decrease over time that was modeled using a first-order regression, thus resulting in higher awareness regarding sample stability or rather instability. All of the samples and calibration standards show the same constant loss rate since they are all on the same plate. Therefore, deferring the measurement time point of a batch up to 30 min has no influence on the calculated AX concentration. However, a delay of more than half an hour may potentially lead to a falsification of the calculated AX values. When measuring with a 10 min delay, an RR of 100 ± 3% could still be determined. Therefore, a 2 min cooling time and 10 min sample transfer time are proposed to achieve the appropriate time interval of a maximum of 12 min. The use of AX as an external calibration standard instead of arabinose or xylose showed no improvement to the calculated AX concentration of the control standards. For time and cost saving, xylose was used as a homogeneous calibration standard material. After parameter adjustment was completed, more than 90% of the analyzed samples (*n* = 50) reached the RR in an appropriate range. The increase in replications due to the use of a 96-well plate provides greater statistical robustness; in addition, even up to 40% of the previous analysis time could be saved. Moreover, the number of individual samples per batch could be significantly increased while accuracy remained at a constant level (RR = 100%). Beer samples of different chemical compositions were analyzed, showing AX concentrations between 500 and 1100 mg L^−1^. Samples indicating high AX concentrations must be diluted to ensure the accuracy of the method with regard to the calibration range (0–300 mg L^−1^). Thus, a wide concentration range can be covered in fully fermented finished beer. Analyses were performed for different conventional beer styles (e.g., lager, pilsner, wheat beer). For samples with incomplete fermentation, an additional step is required. Interfering hexoses that result in an underestimation of WEAX when analyzing samples with high amounts of limit dextrin and fermentable sugars (e.g., wort) were eliminated by *S. diastaticus* fermentation.

LC-MS was used to obtain more information about the red color of the hydrolysates that is induced by a condensation reaction between phloroglucinol and furfural. The resulting derivative could not be identified, and different condensation products were discussed. The red/pink color of the samples probably results from a complex mixture of different hydrolysis products. C_16_H_12_O_6_ was suggested as one potential component formed during hydrolysis which contributes to the coloring. It consists of one molecule of phloroglucinol and two molecules of furfural (*m*/*z* 301 Da). The decrease in absorbance over time, especially at 550 nm, could be confirmed, as the elution profiles of consecutive measurements of the same sample changed considerably. Therefore, a simultaneous measurement of all standards and samples is important. For a large number of samples, this can only be accomplished using a multi-mode microplate reader. Although the measurement device has a slightly higher initial cost, it can be used for a variety of analysis methods in large and small breweries for wort and beer samples.

In this study, a high-throughput application for WEAX quantification was developed and validated in view of three decisive aspects: time, by saving up to 20 min per batch; costs, by reducing the use of resources (i.e., chemical materials); and amount, by significantly increasing the sample number per batch and day. Thus, the advanced Douglas assay is primarily suitable for rapid AX quantification in different beer styles. In addition, molecule structures should be considered more closely to improve filtration performance and reduce related problems. For this purpose, profound studies on the influence of the molecular weight and polymer structure of AX should be performed.

## Figures and Tables

**Table 1:** Possible condensation reactions between phloroglucinol (P) and furfural (F) during heat-induced acid hydrolysis.

Formula	Phloroglucide Derivatives	Molecular Weight	Reference
C_22_H_16_O_8_	2P + 2F − 2H_2_O	409 Da	Goodwin and Tollens [44]
C_11_H_8_O_4_	P + F − H_2_O	205 Da	Jäger and Unger [45]; Goodwin and Tollens [44]
C_11_H_6_O_3_	P + F − 2H_2_O	187 Da	Mann et al. [46]; Kröber [47]
C_16_H_12_O_6_	P + 2F − H_2_O	301 Da	Councler [48]; Leach and Winton [49]
C_17_H_14_O_7_	2P + F − H_2_O	331 Da	Foo and Hemingway [50]
C_17_H_12_O_6_	2P + F − 2H_2_O	313 Da	Chase [28]

## Data Availability

Data are contained within the article.
